# The prediction of occupational health risks of benzene in the printing industry through multiple occupational health risk assessment models

**DOI:** 10.3389/fpubh.2022.1038608

**Published:** 2022-12-14

**Authors:** Bin Shi, Shibiao Su, Cuiju Wen, Tianjian Wang, Haijuan Xu, Ming Liu

**Affiliations:** Guangdong Province Hospital for Occupational Disease Prevention and Treatment, Guangzhou, China

**Keywords:** occupational health, risk assessment, benzene, printing industry, occupational hygiene management

## Abstract

**Background:**

Benzene poisoning is a common occupational poisoning event in the printing industries. Up to now there is still a lack of research data on risk assessment of benzene operations in enclosed workshops. It is crucial to assess the risk level of these positions and put forward effective measures and suggestions.

**Methods:**

The information of selected companies and air samples were collected through on-site investigation, data collation and sample testing were carried out according to the requirements of Chinese standards. The Control of Substances Hazardous to Health (COSHH) Essential, the EPA non-carcinogenic risk assessment model, the Singapore exposure index method and the Chinese semi-quantitative risk assessment models were used to assess the risks of benzene.

**Results:**

The exposed groups all worked more than 8 h per day, and the cleaning, pasting, and packaging groups used general ventilation rather than local ventilation. 28.6% of the printing group and 16.7% of the pasting group had benzene concentrations that exceeded the permissible concentration-time weighted average (PC-TWA) in China. Over 60.0% of the work groups were evaluated at high risk and over 20% of the work groups were evaluated at high cancer risk by the risk assessment models.

**Conclusion:**

The Chinese exposure index method and the synthesis index method may have a stronger practicability. The printing and pasting groups may have a higher risk for benzene exposure. It is necessary to increase protective measures and strengthen occupational hygiene management to reduce risks.

## Background

Benzene is a colorless, sweet and transparent liquid at room temperature. Benzene is considered as a volatile organic compound (VOC) because of its boiling point of 80.1°C and saturated vapor pressure of 12.66 kPa at 25°C (1). Benzene is widely used as fuel and solvent, while it can also be used to synthesize chemical substances. As an important basic raw material of petrochemical industry, benzene is widely used in various industries. At present, the ink used in the packaging and printing industry usually uses benzene as an organic solvent (2).

The primary exposure route of benzene is inhalation. The main health hazard of occupational exposure to benzene is acute poisoning. Data showed that occupational diseases in the printing industry were mostly sporadic with various types, such as benzene poisoning, n-hexane poisoning, toluene poisoning, methanol poisoning, etc. Benzene poisoning is the most common one (3). It was reported that a glue brush worker in a wine box printing plant died of aplastic anemia after working in a high benzene concentration environment for 4 months. In a printing equipment factory, pure benzene was used as a rubber solvent to produce printing rubber cloth. From 1989 to 1994, 33 people were exposed to pure benzene. The prevalence rate of benzene poisoning was 15.2 and 33.3% of the 33 observed objects were benzene poisoning (4). In a private printing plant, a worker who had been working in printing and laminating and had been exposed to adhesives and thinners containing benzene was diagnosed with severe aplastic anemia after 2 years when he developed headaches, bleeding gums, and petechiae on the skin and mucous membranes (5). Several occupational health risk assessment (OHRA) models have been developed to assess health risks from occupational hazards and provide control measures. OHRA not only estimates the likelihood and extent of hazard occurrence through qualitative or quantitative assessments, but also takes appropriate measures to minimize occupational risks (6). Currently, the U.S. Environmental Protection Agency (EPA), Singaporean, Australian, Romanian, International Council of Mines and Metals (ICMM) and UK Basic Model for The Control of Hazardous Substances to Health (COSHH) are considered to be the six most common OHRA models, with the Singapore model having good overall compatibility (7). In 2017, China issued the Occupational health standard “Guidelines for Occupational Health Risk Assessment of Chemicals in the Workplace” (GBZ/T 298-2017) (8), which introduced the basic definition, content and specifications for the use of OHRA. The principles of qualitative risk assessment model are the same as that of COSHH Essential model (9, 10). The semi-quantitative risk assessment models in China include three risk assessment methods: exposure ratio method, exposure index method and synthesis index method. The qualitative and quantitative models in the standard are based on the same principles as the COSHH basic model and the EPA model, respectively. The exposure ratio method, the exposure index method and the synthesis index method were included in the semi-quantitative models. The exposure ratio method and the Singapore model had the same principle, while the exposure index method and synthesis index method are further developed based on the Singapore model (11).

Considering the health and toxic effects of benzene, some industries are gradually adopting other raw materials as substitutes for benzene. However, as benzene is also an excellent chemical solvent, itis still used by many enterprises in China, especially in the printing industry in Shenzhen, China, where cases of benzene exposure poisoning or death remains (12). In this study, multiple OHRA models (COSHH, EPA, Singapore and semi-quantitative risk assessment models) were used to assess the occupational health risks of benzene in China's printing industry. In view of this phenomenon, this study puts forward risk management strategies to reduce the risk of benzene exposure.

## Materials and methods

### Description of the similar exposure groups

Thirty enterprises in the printing industry in Shenzhen, Guangdong Province, China were selected as the research objects. Among these enterprises, benzene is one of the most widely used chemicals. Eight enterprises used benzene as an organic solvent. SEGs were divided into printing groups, cleaning groups, paste groups and packaging groups according to different production processes. In the selected enterprises, the numbers of the four groups are 7, 3, 6, and 3, respectively. The working time, benzene usage, benzene exposure time, process automation, first aid facilities, ventilation, emergency rescue measures, personal protective equipment, occupational health management and benzene concentration of workers in each group were investigated.

### Site survey and on-site testing

Through on-site testing, information on the number of employees, working hours, daily usage of benzene, exposure time, engineering protective measures, personal protective equipment were collected. The collection of air samples and the testing of laboratory benzene samples were performed according to the methods described in Chinese Standards “Specifications of air sampling for hazardous substances monitoring in the workplace (GBZ159-2004)” (13) and “Workplace air aromatic hydrocarbon compounds determination Method” (GBZ/T 160.42-2007) (14). The 8 h time-weighted average concentration (C-TWA) and short-term exposure concentration (C-STEL) were tested. According to the Chinese standard requirements “Occupational Exposure Limits for Hazardous Agents in the workplace Part 1: Chemical hazardous agents (GBZ 2.1-2019)” (15), the permissible concentration-time weighted average (PC-TWA) of benzene is 6 mg/m^3^, and the permissible concentration-short-term exposure limit (PC-STEL) should be less than twice the PC-TWA.

### Occupational health risk assessment models

The China's Occupational health Risk Assessment Guide for Workplace Chemicals (GBZ/T298-2017), COSHH Essential model, EPA model (including non-carcinogenic model and carcinogenic model), Singapore model and domestic semi-quantitative risk assessment model were used to assess the occupational health risk of Benzene. The rationale of these models had been described in detail in the literature and was briefly described as follows.

### The COSHH essential model

This model provided a risk assessment by both exposure levels and health hazards of chemicals. Health hazards were determined by the range of occupational exposure limits (OELs) or by assigning the assessed substance to a hazard band using a Risk-phrase. The exposure level was determined by the physical property and the use of substance.

### The EPA models

These models include non-carcinogenic and carcinogenic risk assessment models. The non-carcinogenic risk is calculated by Equation (1):


(1) $$\begin{array}{l} HQ=\frac{EC}{RfC}. \end{array}$$

Where EC represents the exposure concentration for the acute exposure period, RfC represents the reference concentration for inhalation toxicity (mg/m3), and HQ represents the hazard quotient, which is the value of the non-carcinogenic risk. EC equals to the chemical concentration in the air of the workplace (mg/m3). When the value of HQ is ≥1, it indicates that the toxic and harmful chemicals have a relatively high non-carcinogenic risk (unacceptable risk). Conversely, if the value is lower than 1, it indicates that the toxic and harmful chemicals have a relatively low non-carcinogenic risk (acceptable risk).

Cancer risk is calculated by Equation (2):


(2) $$\begin{array}{l} IR=IUR\times d\times \frac{t_{E}}{t_{L}} \end{array}$$

In the above formula, IUR represents the inhaled unit risk (m3/μg), estimated lifetime cancer risk upper limit from continuous exposure to 1 μg/m3 airborne chemical, D represents the exposure dose to airborne chemical concentration (μg/m3), tL represents life expectancy (a), and tE represents the exposure time (A). tE can be calculated by the following formula: tE = (number of hours per workday x number of workdays per year x duration) / 24 h per day / 365 days per year. When the value of IR is >10^−4^, toxic and hazardous chemicals have a relatively high cancer risk (unacceptable risk). Conversely, when the value is lower than 10^−4^, the toxic and hazardous chemical has a relatively low cancer risk (acceptable risk).

### The Singapore exposure index method

The risk level can be calculated by the equation: $sk=\sqrt{HR\times ER}$, where HR represents the hazard rating, and the ER represents the exposure rating).

In this formula, the HR value can be determined by the carcinogenicity classification determined by the American Conference of Governmental Industrial Hygienists (ACGIH) and the International Agency for Research on Cancer (IARC), or by the median lethal dose (LD50) and median lethal concentration (LC50) of the chemical in the Material Safety Data Sheet (MSDS). The ER was calculated using equation: ${\left[EI_{1}\times EI_{2}\times \cdot \cdot \cdot EI_{n}\right]}^{1/n}$, where EI represents the exposure index, and n represents the number of exposure factors, such as hazard control measures, weekly usage of the chemicals, particle size, vapor pressure, and duration of work per week.

### The semi-quantitative risk assessment model in China

The exposure ratio method, exposure index method and synthesis index method were included in this method. The calculation method of risk level was the same way as the Singapore exposure index method.

In the exposure ratio method, the ER was determined by the ratio of the exposure level (E) and OEL, and the E was calculated using the equation: *E* = *F*×*D*×*M*/*W* (F = the frequency of exposure per week, M = the magnitude of exposure, W = the average working hours per week, and D = the average duration of each exposure). The EI of the Chinese exposure index method takes into account more factors, such as occupational health management, emergency rescue measures, first aid facilities, PPE, daily usage of chemicals and daily working hours. The ratio of exposure level to OEL (E/OEL) was added to the EI in the synthesis index method.

### Statistical analysis

Statistical analysis was performed by SPSS 22.0 software (IBM, Armonk, NY, USA). There were statistical significance of differences between the groups, which was determined by one-way analysis of variance (ANOVA) and the Tukey *post-hoc* test. Cohen's Kappa was used to assess the consistency of the two occupational health risk assessment models (k < 0.40, indicating lack of consistency, 0.75 > k ≥ 0.40, indicating average consistency, k ≥ 0.75, indicating good consistency).

## Results

### On-site survey and test results

The usage of benzene, exposure time of benzene, emergency rescue measures, first aid facilities, process automation, control facilities, occupational health management, benzene concentration of SEG and other information were listed in Table 1. According to the on-site investigation results, the cleaning group and the printing group had relatively high degree of automation, while more than half of the processes in the pasting group and the packaging group were manually operated. The printing groups of many enterprises were equipped with partial ventilation facilities, while the cleaning group, the pasting group and the packaging group were usually equipped with integrated ventilation facilities. Most of companies provided the personal protective equipment for their workers, but a few workers did not wear them at work. Only a few companies were equipped with first-aid facilities, and most of them had poor or lacking occupational health management. The cleaning group and the sticking group had poor emergency rescue measures, and the utilization rate of personal protective equipment was low as well. The benzene concentration in cleaning group and packaging group was significantly lower than the other two groups. The average C-TWA of benzene in printing groups was 4.67 mg/m3, ranging from 0.02 to 24.08 mg/m3. The C-TWA of benzene in cleaning groups were all below 0.02 mg/m3. The average C-TWA of benzene in pasting groups was 3.47 mg/m3, ranging from 0.02 to 20.73 mg/m3. The C-TWA of benzene in packaging groups were all below 0.02 mg/m3. Both the average values of C-STEL and C-TWA in the printing groups were higher than those in the pasting groups. The results of this study showed that C-TWA of 28.6% of the printing group, 16.7% of the paste group and 33.3% of the packaging group exceeded the PC-TWA in the Chinese standard.

**Table 1:** Survey results of SEGs exposed to benzene.

**SEG**		**Printing group**	**Cleaning group**	**Pasting group**	**Packaging group**
Number of group		7	3	6	3
Number of workers per group		4–30	5–6	5–84	6–44
Duration of work (months)		200 (160–240)	184 (160–200)	196 (160–240)	197 (160–240)
Daily usage (kg/L)		104.7 (6–2181)	68.0 (10–118.2)	523.1 (7–3052.5)	14.3 (0.5–30.3)
Weekly usage (kg/L)		596.8 (24–870)	331.9 (50–491)	2628.9 (35–15262.5)	85.4 (2.5–181.8)
Hours of work per day		8.7 (8–10)	8.7 (8–10)	8.7 (8–10)	8.7 (8–10)
Days of work per week		5.3 (5–6)	5.3 (5–6)	5.7 (5–6)	5.7 (5–6)
C-TWA (mg/m^3^)		4.67 (< 0.02–24.08)	< 0.02	3.47 (< 0.02–20.73)	< 0.02
C-STEL (mg/m^3^)		< 0.07			< 0.07
E/OEL		0.78 (0.003–4.01)	0.003	0.58 (0.003–3.45)	0.003
**Result**					
	C-TWA disqualified	28.6% (2/7)	0 (0/3)	16.7% (1/6)	0 (0/3)
	C-STEL disqualified	0			
**Automation level**					
	Full automation	57.2% (4/7)	0 (0/3)	16.7% (1/6)	33.3% (1/3)
	Semiautomation	0 (0/20)	66.7% (2/3)	33.3% (2/6)	0 (0/3)
	Manual operation	42.8% (3/7)	33.3% (1/3)	50.0% (3/6)	66.7% (2/3)
**Ventilation**					
	General ventilation	57.2% (4/7)	100% (3/3)	83.3% (5/6)	33.3% (2/6)
	Local exhaust ventilation	57.2% (4/7)	0% (0/3)	16.7% (1/6)	0 (0/3)
First-aid facility equipped		42.8% (3/7)	33.3% (1/3)	33.3% (2/6)	100% (3/3)
**Personal protective equipment**					
	Equipped	85.7% (6/7)	100% (3/3)	83.3% (5/6)	100% (3/3)
	Used or worn	85.7% (6/7)	66.7% (2/3)	66.7% (4/6)	100% (3/3)
Emergency rescue measures complete		42.8% (3/7)	33.3% (1/3)	33.3% (2/6)	100% (3/3)
**Occupational health management**					
	Performs well	42.8% (3/7)	66.7% (2/3)	50.0% (3/6)	100% (3/3)
	Performs poorly	57.2% (4/7)	33.3% (1/3)	50.0% (3/6)	0 (0/3)
	Lack of management	0 (0/20)	0 (0/3)	0 (0/6)	0 (0/3)

C-STEL, short-term exposure concentration; C-TWA, 8-h time weighted average concentration; E/OEL, the ratio of exposure concentration to the occupational exposure limit; the results here represent the larger ratios of C-TWA/PC-TWA and C-STEL/PC-STEL; PC-TWA, the permissible concentration-time weighted average; PC-STEL, the permissible concentration-short term exposure limit; SEG, the similar exposure group.

### Risk assessment results

As illustrated in Table 2, the risk level of benzene was R45, which indicated a risk of carcinogenic effect on the human body. Therefore, in the COSHH model, hazard level could be classified as grade E. The COSHH Essential model showed that all the working groups exposed to benzene had high risk. The EPA's non-carcinogenic risk assessment model showed that HQs of both the printing groups and the paste groups were >1, indicating that these groups had high non-carcinogenic risk. At the same time, the cancer risk of the printing groups and the pasting groups were 0.004 and 0.003, respectively. Some of the two groups were assessed to be at high carcinogenic risk, accounting for 42.9 and 33.3%, respectively.

**Table 2:** Evaluation results of the COSHH essential model and the EPA models of benzene.

**SEG**	**Number** **of group**	**COSHH essential** **model**			**EPA non-carcinogenic risk** **assessment model**		**EPA carcinogenic risk** **assessment model**		
		**HR**	**ER**	**Risk level**	**HQ**	**Risk level**	**IR**	**Unacceptable risk ratio**	**Acceptable risk ratio**
Printing group	7	E	3	4 (Very high risk)	31.66 (0.17–220.64)	Unacceptable risk (42.9%)	1.45 × 10^−3^ (2.9x10^−6^-0.01)	42.9% (3/7)	57.1% (4/7)
Cleaning group	3	E	3	4 (Very high risk)	0.19–0.66	Acceptable risk (100.0%)	2.14 × 10^−5^ (2.9 x10^−6^-4x10^−5^)	0	100% (3/3)
Pasting group	6	E	3	4 (Very high risk)	2.88 (0.02–17.04)	Unacceptable risk (33.3%)	1.67 × 10^−3^ (2.9x10^−6^-0.01)	33.3% (2/6)	66.7% (4/6)
Packaging group	3	E	3	4 (Very high risk)	0.23	Acceptable risk (100.0%)	6.55 × 10^−6^ (2.9 × 10^−6^-1 × 10^−5^)	0	100% (3/3)
Total	19	E	3	4 (Very high risk)	12.68 (0.02–220.64)	Unacceptable risk (26.3%)	1.07 × 10^−3^ (2.9 × 10^−6^-0.01)	26.3% (5/19)	83.7% (14/19)

COSHH, UK Control of Substances Hazardous to Health; EPA, U.S. Environmental Protection Agency. ER, exposure rating; HR, hazard rating. HQ, the hazard quotient; IR, the excess personal risk of carcinogenic inhalation; SEG, the similar exposure group.

According to the IARC, benzene can be classified as a class 1 substance, also known as a confirmed carcinogen in humans. The HR of benzene can be divided into five levels in the semi-quantitative risk assessment model. As shown in the exposure index method results, the risk levels of each working groups ranged from 2 to 5. 28.6% of the printing groups, 16.7% of the pasting groups and 33.3% of the packaging groups were at very high risk. The Singapore exposure index method showed that the risk levels of the work groups were distributed from grade 4 to 5. The Singapore exposure index method showed that the risk levels of the work groups were distributed between 4 and 5, with 84.2% of the work groups at very high risk, including 71.4% of the printing group, 100% of the cleaning group, 100% of the pasting group, and 66.7% of the packaging group. The China exposure index method showed that the risk levels of the working groups ranged from 3 to 5, and 73.7% of the work groups were at high risk, which were 75.8% of the printing groups, 100% of the cleaning groups, 66.7% of the pasting groups, and 33.3% of the packaging groups. Only one of the pasting groups was at very high risk. 21.1% of the work groups were at medium risk, including 14.3% of the printing groups, 16.7% of the paste groups and 66.7% of the packaging groups. The synthesis index method showed that the risk levels of the work groups were distributed from 2 to 4, among with 75.8% of the printing groups, 66.7% of the cleaning groups, 66.7% of the paste groups bring at high risk, and the risk grade of the packaging groups being at medium or low (Table 3).

**Table 3:** Evaluation results of semi-quantitative risk assessment models of benzene.

**SEG**	**Number of group**	***R***	**exposure ratio method**	**Singapore exposure index method**	**Chinese exposure index method**	**Synthesis index method**
Printing group	7	2	71.4% (5/7)	0	0	0
		3	0	0	14.3% (1/7)	14.3% (1/7)
		4	0	28.6% (2/7)	75.8% (6/7)	75.8% (6/7)
		5	28.6% (2/7)	71.4% (5/7)	0	0
Cleaning group	3	2	100.0% (3/3)	0	0	0
		3	0	0	0	33.3% (1/3)
		4	0	0	100% (3/3)	66.7% (2/3)
		5	0	100% (3/3)	0	0
Pasting group	6	2	83.3% (5/6)	0	0	0
		3	0	0	16.7% (1/6)	33.3% (2/6)
		4	0	0	66.7% (4/6)	66.7% (4/6)
		5	16.7% (1/6)	100% (6/6)	16.7% (1/6)	0
Packaging group	3	2	66.7% (2/3)	0	0	33.3% (1/3)
		3	0	0	66.7% (2/3)	66.7% (2/3)
		4	33.3% (1/3)	33.3% (1/3)	33.3% (1/3)	0
		5	0	66.7% (2/3)	0	0
Total	19	2	78.9% (15/19)	0	0	5.3% (1/19)
		3	0	0	21.1% (4/19)	31.6% (6/19)
		4	5.3% (1/19)	15.8% (3/19)	73.7% (14/19)	63.2% (12/19)
		5	15.8% (3/19)	84.2% (16/19)	5.3% (1/19)	0

R, risk level; SEG, the similar exposure group.

According to the available literature (16), the WBC counts of workers exposed to low concentrations of benzene did not change significantly over time, except when benzene concentrations were relatively high. In this study, the cleaning and packaging groups were exposed to low concentrations of benzene, while the printing and pasting groups were exposed to relatively high concentrations of benzene, and thus had a higher occupational health risk.

The consistency of bidirectional ordinal classification data was evaluated by the Cohen's Kappa generally. As shown in Table 4, there was a lack of consistency between the exposure ratio method and all three methods. Furthermore, there was a lack of consistency between the Singapore Exposure Index method and the Chinese Exposure Index method, as well as between the Singapore Exposure Index method and the Synthesis Index method. In addition, there was general consistency between the Chinese exposure index method and the Synthesis index method.

**Table 4:** Cohen's Kappa results of semiquantitative risk assessment models of benzene.

**Cohen's Kappa (A vs. B)**	**Value**	**Approx. Sig**.
Exposure ratio method vs. Singapore exposure index method	0.019	0.656
Exposure ratio method vs. Chinese exposure index method	0.027	0.597
Exposure ratio method vs. Synthesis index method	−0.013	0.845
Singapore exposure index method vs. Chinese exposure index method	−0.066	0.243
Singapore exposure index method vs. Synthesis index method	−0.052	0.243
Chinese exposure index method vs. Synthesis index method	0.438	0.018

## Discussion

The COSHH Essential model, (EPA model) and semi-quantitative risk assessment model (Singapore model and China semi-quantitative risk assessment model) were used to assess occupational health risk of benzene in this study. Each occupational health risk assessment model has its own advantages and disadvantages (6, 7, 17). The COSHH Essential model is mainly used for small and medium-sized enterprises. This method is relatively simple and easy to operate, but sometimes it would overestimate the risk level and make a possible deviation. The strengths and weaknesses of the EPA model are equally apparent. As a quantitative assessment model, this model can fully assess the non-carcinogenic and carcinogenic risks of chemicals, and its reference concentration (Rfc) and inhalation unit risk (IUR) are determined based on epidemiological and toxicological data. However, the EPA model also has some shortcomings. For instance, the model can not assess the chemicals lack of Rfc and IUR values. In addition, for different risk levels, the model is also difficult to distinguish between different risk levels, and the results can only be expressed as “high” and “low.” Semi-quantitative risk assessment models are based on semi-quantitative calculations, using both quantitative and qualitative methods. The Exposure Ratio Method focuses on the exposure level of chemical substances, and the exposure index method is used when there is a lack of air monitoring data. The Singapore Exposure Index Method is evaluated by steam pressure or particle size, chemical dosage, working hours and hazard control measure, while the Chinese exposure index method has a higher exposure index than the Singapore exposure index method, including personal protective equipment, first aid facilities, emergency rescue facilities, occupational health management, etc. The composite index method considers not only the exposure level, but also all exposure indicators. The disadvantage of the semi-quantitative risk assessment model is that the classification of the exposure indices is relatively rough.

In all enterprises involved in this study, C-TWA of benzene in printing group and pasting group exceeded the occupational exposure limits, while the C-TWA in the packaging group and the cleaning group is relatively low. This is due to the higher chemical use in the printing and bonding groups, insufficient local ventilation, and relatively poor occupational health management.

The results of the COSHH Essential model showed that all working groups were at very high non-carcinogenic risk, while the EPA non-carcinogenic risk assessment model showed a high non-carcinogenic risk for the printing and paste groups. In the COSHH Essential model, since the HR of benzene was E, the principle of the model states that the risk level is 4 (very high risk) regardless of the exposure level. The RfC in the EPA's model of carcinogenic risk assessment represents the reference concentration at which sustained inhalation would not result in a lifetime health risk. Because of the low RfC of benzene, the risk level remained high even when the detected concentration was below the detection limit. In fact, low concentrations of benzene exposure (< 0.5 OEL), high levels of automation, good ventilation, good emergency response, good management, and high use of personal protective equipment in some industries could reduce the risk. In this case, the EPA's non-carcinogenic risk assessment model and the COSHH Essential model generally overestimated the risk level of exposure to benzene in the working group. The results of the semi-quantitative risk assessment model indicated that the working group's risk levels ranged from 2 to 5. In the Exposure Ratio Method, the level of risk was only related to the concentration of exposure, ignoring the effect of protective measures. The Chinese exposure index method and the Singapore exposure index method focus on exposure factors other than exposure concentration. The Chinese Exposure Index Method focused on more exposure factors compared with the Singapore Exposure Index Method, such as personal protective equipment, emergency rescue measures, first aid facilities, occupational health management, etc. Based on the Chinese exposure index method, the synthesis index method added exposure concentration as another exposure factor. The results showed that the evaluation results of Singapore exposure index method were higher than those of China exposure index method and comprehensive index method, while the evaluation results of the other two methods for these four working groups were basically the same. According to the actual situation of each working group, the lower the exposure concentration of benzene, the more effective the hazard control measures, the better the emergency rescue facilities, the more sound the occupational health management, and the lower the risk will be. To sum up, the Chinese exposure index method and synthesis index method were relatively more practical. At the same time, since occupational health management and engineering control measures may affect the concentration of chemicals in the workplace, the exposure factors to be considered by the integrated index method should be carefully chosen in order to avoid bias. Results from the EPA cancer risk Assessment model showed that nearly half of the working groups within the printing and paste groups in the printing industry were assessed as having a high risk of cancer. Cancer risk levels tended to be lower only when benzene doses were lower and work hours were shorter. China is one of the industrial power in the world. In the traditional occupational health assessment, the assessment of occupational health hazards of benzene has always been in line with the national health standards. When the concentration of benzene exposure is lower than the national health standards, it is considered as a safe operation. A study by Lan et al. (18) showed that workers exposed to 1 ppm (3.19 mg/m3) benzene showed homosexuality and impaired hematopoietic stem/progenitor cell self-renewal. In other words, the risk of benzene exposure is still high and can cause health damage to workers even at low dose levels, which is consistent with the assessment results of this study. Huang et al. developed a model for cancer risk assessment of benzene exposure based on a physiological toxicokinetic model and a dose-response relationship model using a benzene exposure cohort population in collaboration with the Chinese Society for Preventive Medicine and the American Institute for Cancer Research (19, 20). He also found that the predicted cancer risk for workers at exposure concentrations of 50–500 mg/m3 ranged from 1.52 × 10^−4^ to 1.19 × 10^−3^, which was higher than the maximum acceptable risk value and consistent with the actual cancer incidence rate. Therefore, it is recommended that the health administration departments carry out the risk assessment of carcinogenic and non-carcinogenic effects of benzene along with the occupational health risk assessment of benzene to promise a safe working environment for workers.

According to the results of this risk assessment, most of the working groups in the printing industry that are exposed to benzene are at high risk, with higher exposure risks in the printing and paste groups. Due to the high occupational health risk of benzene in the printing industry in China, risk management measures should be carried out. Enterprises should optimize and reform the operating conditions. For high-risk jobs, risks should be reduced according to the priorities of replacement, improved design, isolation, administration and personal protection. Benzene should be replaced by non-toxic toluene and ethanol should be used as organic solvents or extraction agents. The production process should be sealed, automated, programmed, and regularly maintained. The workplace should contain sufficient local ventilation and detoxification equipment. In addition, occupational health training is arranged regularly to raise workers' awareness of self-protection and make them wear gas masks voluntarily. Regular medical checkups should be conducted for workers, and workers should be immediately stopped from the position once they are diagnosed with low white blood cells.

## Conclusion

The OHRA model in Chinese standard GBZ/T 298-2017 can be used for occupational health risk assessment of benzene. China exposure index method and composite index method are more realistic than the others. The results of the current study indicated that there are many high-risk of benzene exposure in the printing industry in China, and the risk of benzene exposure may be in the printing group and the pasting group. It is necessary to take measures to reduce the risk of benzene exposure in these work positions.

## Data availability statement

The raw data supporting the conclusions of this article will be made available by the authors, without undue reservation.

## Author contributions

BS was responsible for the design of experimental methods, actual research, analysis of experimental data, visualization of experimental results, and writing of the first draft of the paper. SS was responsible for the concept generation, funding acquisition, supervision and guidance, and review and revision of the paper. CW was responsible for the actual research and analysis of experimental data. TW, HX, and ML was responsible for the data collection. All authors contributed to the article and approved the submitted version.
